# Mitigating product inhibition in 2′‐hydroxybiphenyl‐2‐sulfinate desulfinase (DszB) with synthetic glycosylation

**DOI:** 10.1002/pro.70187

**Published:** 2025-06-16

**Authors:** Junbao Liang, Yi Zheng, Valerie Vaissier Welborn

**Affiliations:** ^1^ Department of Chemistry Virginia Tech Blacksburg Virginia USA; ^2^ Macromolecules Innovation Institute Virginia Tech Blacksburg Virginia USA

**Keywords:** 4S pathway, AMOEBA, DszB, glycation, molecular dynamics, polarizable force field

## Abstract

The combustion of sulfur‐rich crude oil is toxic to the environment, making the removal of sulfur impurities a priority for the sustainable use of liquid fuels. Biodesulfurization via the 4S pathway is a promising approach due to its C‐S bond cleavage specificity and mild operating conditions. However, biodesulfurization is not economically viable due to the slow turnover of 2′‐hydroxybiphenyl‐2‐sulfinate desulfinase (DszB), an enzyme catalyzing the conversion of 2′‐hydroxybiphenyl‐2‐sulfinate to 2‐hydroxybiphenyl and sulfite. Previous studies have identified product inhibition as the limiting factor in DszB, whereby solvent‐exposed protein loops obstruct the active site after substrate binding. This closed conformation is stabilized by hydrophobic interactions between the loops and the product. Here, we propose an artificial glycosylation strategy to mitigate product inhibition in DszB. We modeled glycated DszB in the apo, ligand‐bound, and product‐bound states with molecular dynamics based on the AMOEBA polarizable force field, and analyzed the chemical positioning of the reactant and product compared to the wild type (WT). We find that the addition of glucose on three Ser loop residues increases the interaction of the loops with water, overcoming the weaker product–loop interactions, and thereby enabling product release. Importantly, the enhanced flexibility of the loops was subtle enough to not heavily disrupt the chemical positioning of the reactant, which suggests that the rate acceleration would be similar to that of the WT.

## 1 INTRODUCTION

Crude oil, or petroleum, is a liquid fossil fuel that originates from the remains of marine organisms (Onyenekenwa Cyprian Eneh, 2011). Crude oil contains between 50% (heavy crude oil) and 97% (light crude oil) of hydrocarbons (Bello et al., 2021; Malani et al., 2021). The other 50% to 3% fraction consists of compounds containing nitrogen, sulfur, oxygen, and metals, which reduces the market value of the corresponding oil (Feng et al., 2016; Kilbane Ii & Le Borgne, 2004). Indeed, refining crude oil with high sulfur content leads to severe environmental pollution and degradation, as well as corrosion (Dada et al., 2025; Sousa et al., 2020). As resources deplete, more countries will turn to the combustion of heavy crude oil, making desulfurization indispensable to future refinery practices (Mills, 2019; Pham et al., 2024; Yu, 2017).

Hydrodesulfurization is currently the most common method to remove sulfur from petroleum (Bello et al., 2021; Pham et al., 2024). Hydrodesulfurization is a process that relies on high temperature and pressure to break reactive C–S bonds, releasing sulfur‐free hydrocarbons and hydrogen sulfide (Pham et al., 2024; Shafi & Hutchings, 2000). This process is efficient in desulfurizing inorganic sulfur compounds and thiols. However, conjugated organic sulfur compounds are more stable and resist hydrodesulfurization, even at higher temperatures (Bello et al., 2021; Boniek et al., 2015). Unfortunately, the elemental content of sulfur in crude oil can reach 8 wt% and exist in organic compounds, including thiophenes, benzothiophenes (BT), dibenzothiophenes (DBT), and their derivatives (Malani et al., 2021; Wauquier, 1995).

**FIGURE 1:**
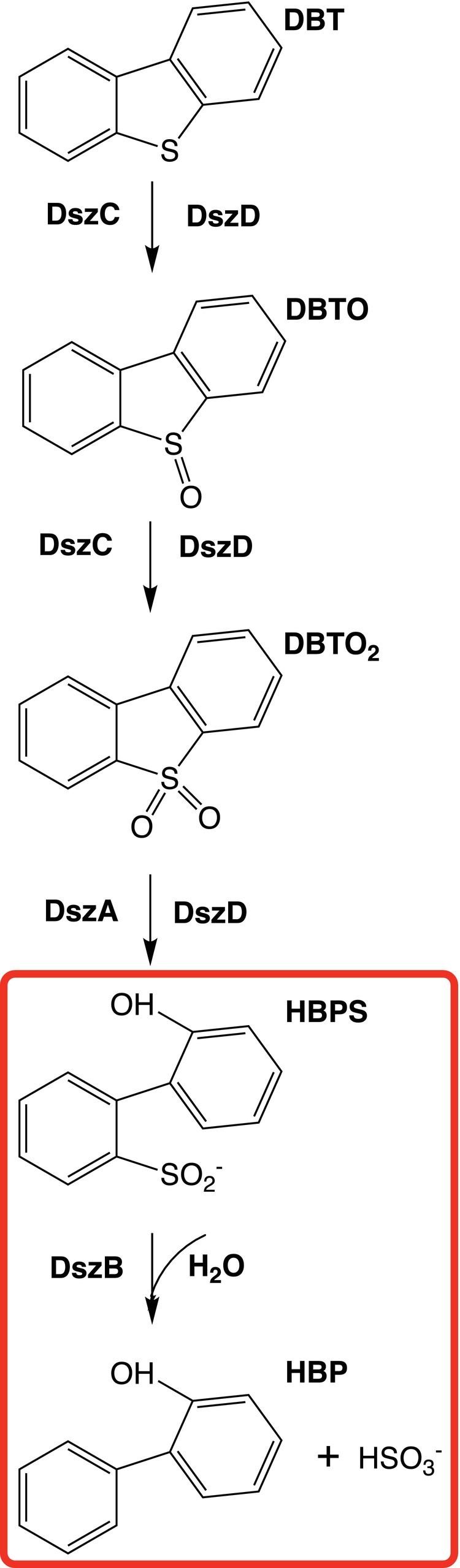
Biodesulfurization 4S pathway catalyzed by enzymes DszA‐D in *Rhodococcus erythropolis IGTS8*. The desulfurization of dibenzothiophene (DBT) occurs in four steps, yielding hydroxybiphenyl (HBP) and sulfite through the formation of dibenzothiophene sulfoxide (DBTO), dibenzothiophene sulfone (DBTO_2_) and hydroxylphenyl benzene sulfinate (HBPS).

Biodesulfurization has the potential to address this issue, using enzymes expressed in microorganisms to catalyze sulfur removal from organic compounds (Boniek et al., 2015; Dudley & Frost, 1994). To date, the most efficient enzymatic pathway for the desulfurization of DBT and its derivatives is the so‐called 4S pathway, catalyzed by enzymes DszA‐D in *Rhodococcus Erythropolis IGTS8* (Feng et al., 2016; Kilbane Ii & Le Borgne, 2004; Sousa et al., 2020). As its name indicates, the 4S pathway involves four sulfur intermediates: DBT sulfoxide (DBTO), DBT sulfone (DBTO2), 2‐(2′‐hydroxyphenyl) benzene sulfinate (HBPS), and sulfite (Figure 1) (Gray et al., 2003; Yu et al., 2019). The 4S pathway preserves the integrity of the hydrocarbon structure, thereby maintaining the caloric value of the fuel. Despite its unique properties, the 4S pathway cannot be used for industrial applications due to its limited efficiency.

One of the major limitations of the 4S pathway for industrial sulfur removal is the low level of expression of the *dsz* genes in bacteria (Martínez et al., 2016; Martínez et al., 2017). Numerous strategies have been proposed over the years to create a genetically improved 4S pathway that would be suitable for industrial applications. The genetic control elements that have shown potential include the cloning of multiple copies of the *dsz* genes, enhancing the expression of the *dsz* genes, modifying the ribosome binding site, and the genetic rearrangement of the *dsz* genes (Kilbane II, 2006; Ma et al., 2006; Martínez et al., 2016; Matsui et al., 2001; Reichmuth et al., 2004; Shavandi et al., 2009; Wang et al., 2011). For example, Li et al. overexpressed a desensitized DszC via directed evolution, saturation mutagenesis, and high‐throughput screening, reporting a 14‐fold increase in catalytic activity (Li et al., 2019). Alternatively, Guo et al. rearranged the dszABC expression ratio from 11:3.3:1 to 1:16:5, which yields a 12‐fold enhancement in catalytic activity (Li et al., 2008).

Another major bottleneck that limits the efficiency of the 4S pathway is product inhibition (Feng et al., 2016; Mills, 2019; Silva et al., 2023; Yu, 2017; Yu et al., 2019). Product inhibition is responsible for slow turnover in enzymatic reactions, which become the rate‐limiting step in metabolic pathways (Han et al., 2016; Wang et al., 2019). In living organisms, product inhibition is beneficial because it is a form of negative feedback that enables metabolic regulation. In industry, however, product inhibition is detrimental, causing low product yields. In the 4S pathway, the product HBP has strong structural similarity with HBPS and the other intermediates, making HBP a competitive inhibitor for DszB (Abin‐Fuentes et al., 2013). Many have tried reducing product inhibition through mutagenesis, modified expression of the dsz operon, etc. For example, Ohshiro et al. used site‐directed mutagenesis on DszB to perform two mutations in the product binding site: Tyr63Phe and Gln65His, gaining a 30% increase in catalytic activity and better thermostability (Ohshiro et al., 2007). Interestingly, they report that single mutations will destabilize the protein (Ohshiro et al., 2007). Overall, it has proven remarkably challenging to engineer mutations that would enhance product release without modifying the kinetics of the catalytic step. To circumvent this issue, Han et al. have introduced conformational flexibility in chorismite‐pyruvate lyase, which has resulted in an 8‐fold reduction in product inhibition and a 3‐fold enhancement in catalytic rate (Han et al., 2016).

**FIGURE 2:**
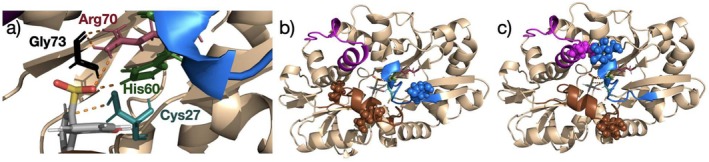
(a) Key active site residues in DszB. (b) Glucose O‐linked to the three Ser residues in DszB's loops. The glucoses are shown in Van der Waals representation, for clarity. There is one glucose/Ser in loop 1 (blue), none in loop 2 (magenta), and two in loop 3 (brown). (c) Glucose O‐linked to the three Thr residues in DszB's loops. The glucoses are shown in Van der Waals representation, for clarity. There is one glucose/Thr in loop 1 (blue), one in loop 2 (magenta), and one in loop 3 (brown).

In this work, we propose an alternative strategy to modulate product inhibition in DszB through enhanced conformational flexibility. In this step, hydrogen atoms in His60 and Arg70 form a hydrogen bond network with the sulfinate group (SO_2_^−^) of HBPS, anchoring it in place (Figure 2a). SO_2_^−^ then reacts with Cys27 to form HSO_3_^−^, yielding the sulfur‐free HBP (Gray et al., 2003; Sousa et al., 2020). In principle, HBP is more flexible than HBPS in the active site as the aforementioned hydrogen bonds cannot form, enabling the release of HBP (Das et al., 2025; Yu et al., 2019). However, DszB undergoes conformational changes upon binding of the substrate (Callender & Dyer, 2006; Gutteridge & Thornton, 2004). The changes are primarily located in three flexible loops located near the active site (Figure 2) (Callender & Dyer, 2006; Gutteridge & Thornton, 2004). Product inhibition occurs when these conformational changes obstruct the active site, trapping substrate and product (Lee et al., 2006; Yu et al., 2019). Despite inherent loop flexibility, the reversal of the conformational changes that obstruct the active site is not favored due to hydrophobic interactions between the loop residues and HBP (Mills, 2019; Yu, 2017; Yu et al., 2019). We conducted a computational study investigating the feasibility of loop glycation to facilitate product release and increase catalytic turnover without reducing the catalytic rate. Specifically, we analyze the influence of a series of O‐linked glucose on the dynamics of the loops responsible for the obstruction of the active site in DszB (Figure 2b,c). The addition of glucose aims to pull the loops toward the solvent and overcome the, presumably weaker, loop–HBP interactions. Our simulations confirm that HBP is more likely to escape in glycosylated DszB and that these posttranslational modifications do not alter the structural integrity of the active site.

## 2 RESULTS AND DISCUSSION

**FIGURE 3:**
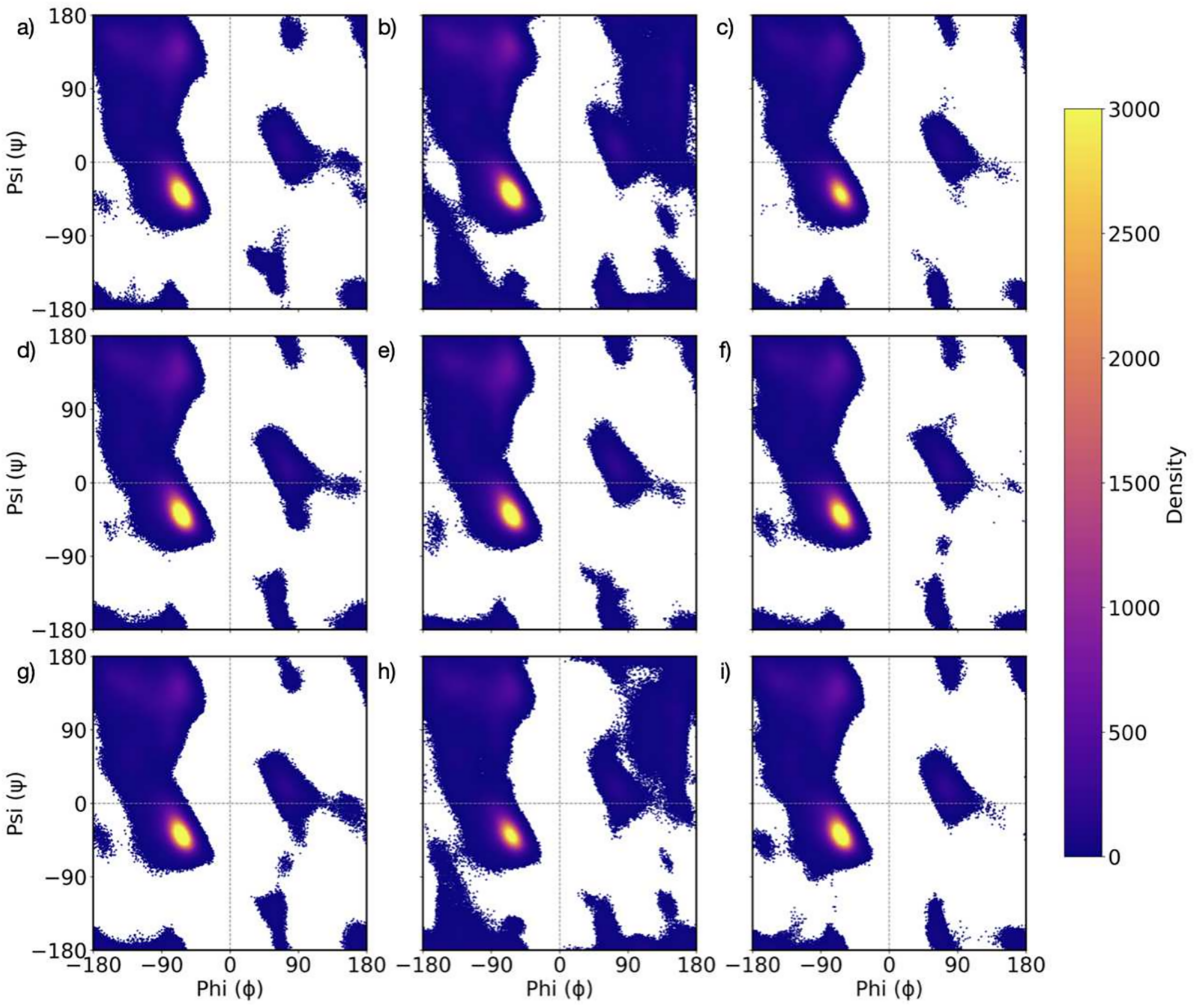
Ramachandran plots showing the distribution of the (ψ,ϕ) dihedral pairs in (a) DszB WT in the apo state, (b) DszB Ser Glc in the apo state, (c) DszB Thr Glc in the apo state, (d) DszB WT in the ligand (HBPS)‐bound state, (e) DszB Ser Glc in the ligand (HBPS)‐bound state, (f) DszB Thr Glc in the ligand (HBPS)‐bound state, (g) DszB WT in the product (HBP)‐bound state, (h) DszB Ser Glc in the product (HBP)‐bound state, (i) DszB Thr Glc in the product (HBP)‐bound state.

To test the influence of loop glycation on product release, we modeled the apo, ligand‐bound, and product‐bound states of DszB wild type (WT), DszB with glucose O‐linked to Ser residues in loops 1 and 3 (Ser Glc), and DszB with glucose O‐linked to Thr residues in loops 1–3 (Thr Glc). As described in Methods, we ran three replicates of 150 ns in each case, for a total of 27 MD simulations. In Figure 3, we show the Ramachandran plots for all residues in the nine aforementioned cases. We observe very similar distributions of the dihedral angle pairs (ψ,ϕ) with higher‐density zones in the middle and top left quadrants. Two plots stand out with additional regions of allowed backbone dihedral pairs, namely, the plots characterizing DszB Ser Glc in the apo and product‐bound states (Figure 3b,h). Note that these areas do not cover known secondary structure zones (e.g., α−helix and β−sheets.), suggesting that the new possible (ψ,ϕ) pairs correspond to new conformations of unstructured segments. These results show that the glycation of Thr residues in DszB does not affect the conformation of the protein backbone at all and that the glycation of Ser residues preserves the structure in the ligand‐bound state while allowing more degrees of freedom for unstructured segments in the apo and product‐bound states. However, as described in Methods, the apo state in Figure 3 is modeled from the crystal structure of the ligand‐bound state. In Figure S1, we compare these with Ramachandran plots of the apo state modeled from a ligand‐free crystal structure. In this case, we observe very similar plots for DszB WT, DszB Ser Glc, and DszB Thr Glc, suggesting that the additional degrees of freedom of unstructured fragments could be a signature of the product‐bound state only.

**FIGURE 4:**
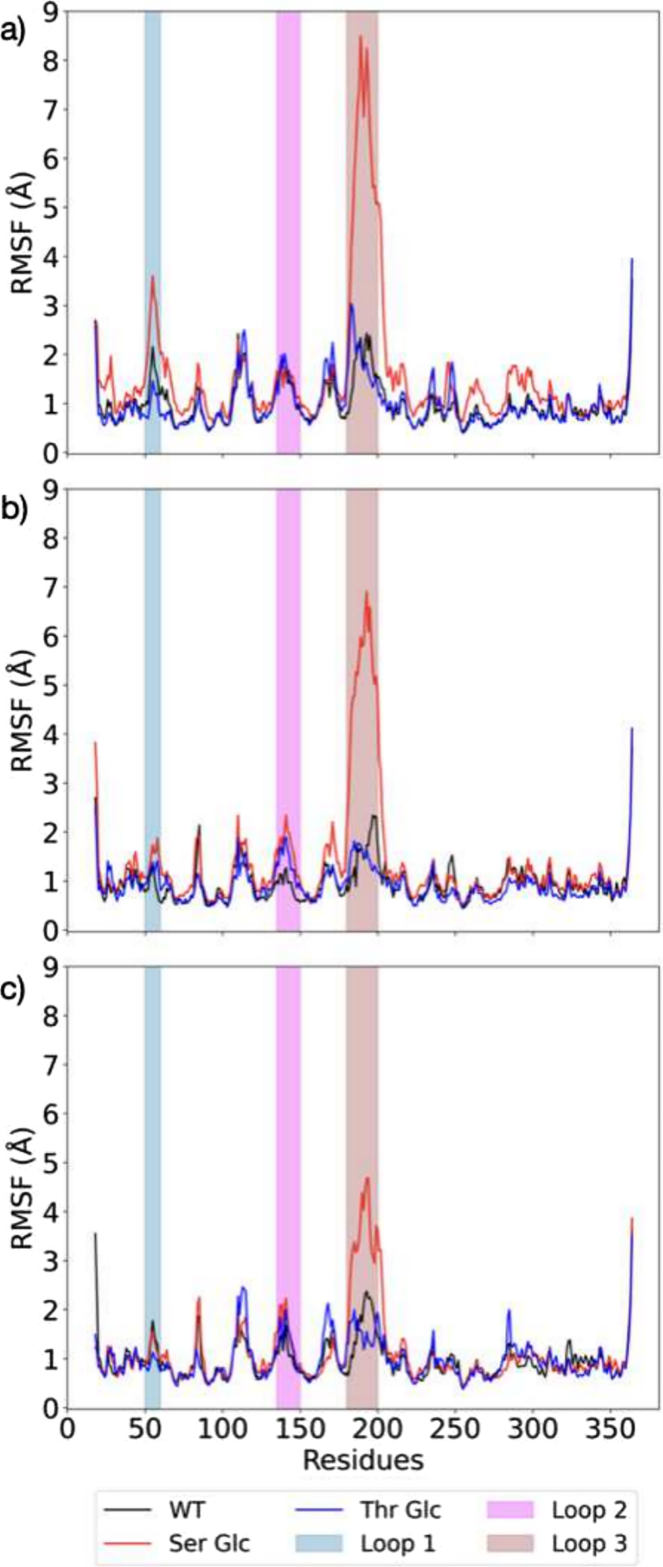
Root mean square fluctuation (RMSF) for DszB WT, Ser Glc, and Thr Glc in the (a) apo, (b) ligand (HBPS)‐bound, and (c) product (HBP) bound states. The residue index range corresponding to the three loops obstructing the active site in DszB is shown in blue (loop 1), magenta (loop 2), and brown (loop 3).

To better understand the relative flexibility of the loops with respect to the rest of the protein, we present in Figure 4 the root mean square fluctuation (RMSF) of each residue in DszB WT, Ser Glc, and Thr Glc in the apo (Figure 4a), ligand‐bound (Figure 4b), and product‐bound states (Figure 4c). The RMSF plot for the second apo state is shown in Figure S2, for reference. For DszB WT, the residues in loop 1 show the greatest flexibility in the apo state with an RMSF over 2 Å. We also note that the RMSF of loops 1 and 2 decreases significantly in the ligand‐bound state. This is consistent with the presence of HBPS in the active site that non‐covalently interacts with the residues in the loops, keeping them in place. Interestingly, the RMSF of the residues in loop 3 of DszB WT looks similar in both the apo and ligand‐bound states, suggesting that loop 3, or part of it, does not interact directly with HBPS. In the product‐bound state, DszB WT exhibits enhanced loop flexibility compared to the ligand‐bound state, especially for loops 1 and 2. However, the RMSF of each loop is less than that of the apo state. This is also consistent with previous work that stated that DszB loops undergo a large conformational change upon substrate binding; changes that are hard to reverse, keeping the product trapped in the active site (Mills, 2019; Yu, 2017).

For Dsz B Thr Glc, with one glycated Thr residue in each loop, we see that the RMSF is very close to that of the WT in all three states. The only exception would be an enhanced loop 2 RMSF for Thr Glc in the ligand‐bound state. For DszB Ser Glc, the RMSF of loop 1 keeps to that of DszB WT in both bound states and exhibits greater flexibility in the apo state. Loop 2 follows a similar pattern in the apo and product‐bound states, with greater flexibility in the ligand‐bound state. Note that DszB does not have a Ser residue in loop 2; therefore, no glucose (see Methods). This implies that the significant change in RMSF observed in the ligand‐bound state likely propagates from changes in the nearby loop 3. In fact, loop 3 in Ser Glc, which has two glucose O‐linked to Ser181 and Ser194, exhibits strong RMSF that reaches 8, 6, and 5 Å in the apo, ligand‐bound, and product‐bound states, respectively. This is one of the key design components of our glycated DszB: a greater loop flexibility to reverse the conformational changes that occurred upon ligand binding and liberate the product from the active site.

**FIGURE 5:**
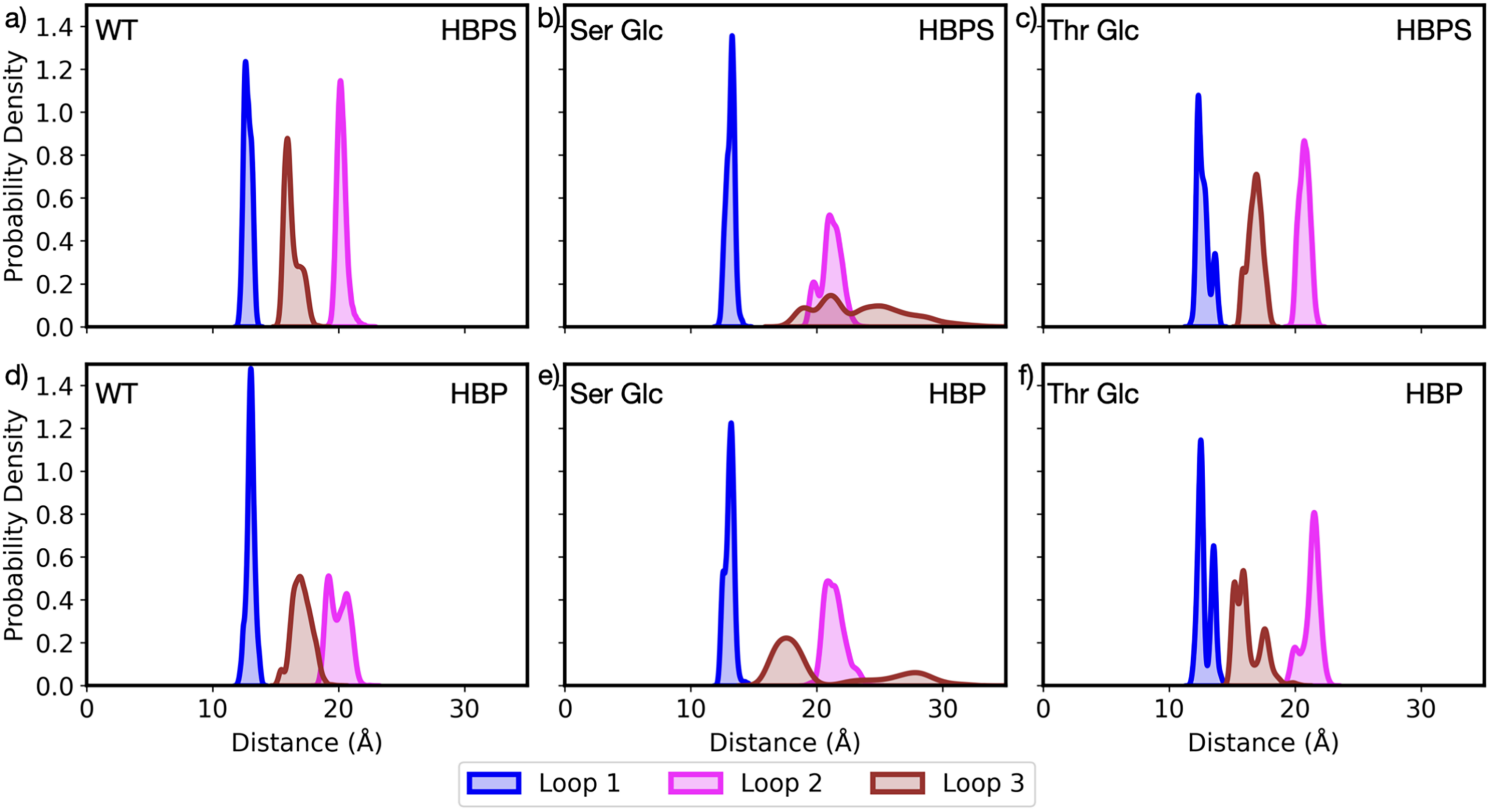
Probability density of the average pairwise distance between the residues in the loops and the active site residues Cys27, Arg70, Gly73, and His60 in (a) DszB WT in the ligand (HBPS)‐bound state, (b) DszB Ser Glc in the ligand (HBPS)‐bound state, (c) DszB Thr Glc in the ligand (HBPS)‐bound state, (d) DszB WT in the product (HBP)‐bound state, (e) DszB Ser Glc in the product (HBP)‐bound state, and (f) DszB Thr Glc in the product (HBP)‐bound state.

To verify that the greater loop flexibility in the product‐bound state resulted in the opening of the active site, we computed the average pairwise distance between the residues in loops and the active site residues (Cys27, Arg70, Gly73, and His60). The corresponding probability distributions are presented in Figure 5 for the ligand‐bound and product‐bound states. We observe that loop 1 (in blue in Figure 5) is the least affected by the nature of the substrate in the active site or by the glycosylation of Ser and Thr residues, exhibiting in each case a narrow distribution around 12–13 Å. In the ligand‐bound state, loops 2 and 3 of DszB WT are characterized by narrow, non‐overlapping distributions centered around 16 and 20 Å, respectively. We observe similar distributions for DszB Thr Glc. In the product‐bound state, the distance distributions of these loops shorten and broaden, but remain mostly separated. However, for DszB Ser Glc in both the ligand‐ and product‐bound states, we see that the distance distributions for loops 2 and 3 flatten and overlap for distances greater than 20 Å. This suggests that these loops are more flexible, visiting more conformations (broad distributions), and are further away from the active site (shifted distributions). This is confirmed by the radial distribution functions between the oxygen of the glycated Ser and Thr residues and that of the water (Figures S3–S5). Indeed we see that these residues are more solvent‐exposed, surrounded by a greater number of water molecules in DszB Ser Glc ligand‐ and product‐bound states. This coincides with an increased disorder of the water molecules around the residues, consistent with a greater conformational flexibility. We also show in Figure S6 a snapshot of the simulations showing the loops in DszB Ser Glc and DszB Thr Glc exposed to water.

**FIGURE 6:**
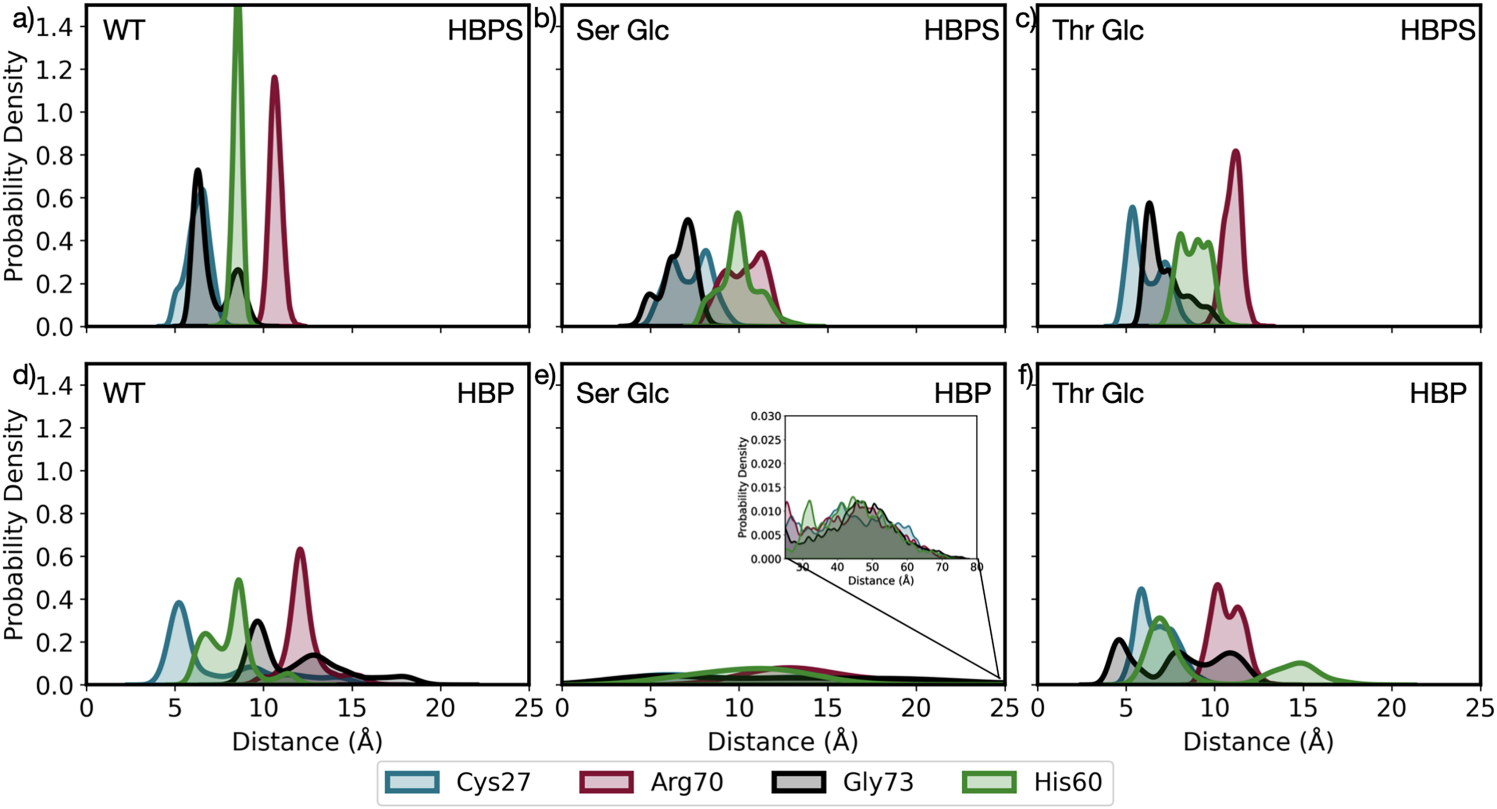
Probability density of the average pairwise distance between the substrate (HBPS or HBP) and the active site residues Cys27, Arg70, Gly73, and His60 in (a) DszB WT in the ligand (HBPS)‐bound state, (b) DszB Ser Glc in the ligand (HBPS)‐bound state, (c) DszB Thr Glc in the ligand (HBPS)‐bound state, (d) DszB WT in the product (HBP)‐bound state, (e) DszB Ser Glc in the product (HBP)‐bound state, and (f) DszB Thr Glc in the product (HBP)‐bound state.

However, for our design to be competitive, we require greater loop flexibility to facilitate product release while preserving the interactions between HBPS and the active site residues Cys27, His60, Arg70, and Gly73 (see snapshot in Figure S7). Therefore, we need to verify that our glycation strategy is associated with product release and does not change the chemical positioning of HBPS, which would interfere with the catalytic activity of the enzyme. Then, we computed the average pairwise distance between the substrate and the active site residues (Figure 6).

In Figure 6a, we see that HBPS is closest to Cys27 and Gly73 (7 Å), followed by His60 (8.5 Å) and Arg70 (11 Å). The probability distributions for Cys27 and Gly73 (His60 and Arg70) are short and broad (tall and narrow), suggesting a flexible (stiff) interaction. In comparison, all distributions for HBP in DszB WT (Figure 6d) are short, broad, and shifted to shorter (longer) distances for Cys27 and His60 (Gly73). This confirms that HBP remains in the active site and engages in non‐covalent interactions with the active site residues. A similar observation can be made for Thr Glc (Figure 6c,f), such that both HBPS and HBP engage in interactions that mimic those of DszB WT in the HBP‐bound state. This means that DszB Thr Glc does not improve the likelihood of product release and potentially compromises chemical positioning in the active site. In Figure 6b, we see that the distributions for DszB Ser Glc in the HBPS‐bound state are short and broad, but centered around the same distances as DszB WT. This suggests that while the binding pose of HBPS seems more flexible in DszB Ser Glc than in DszB WT, the reactant comes into contact with the active site residues enough to react. We further validate the precise chemical positioning of HBPS in the active site by computing the distance between the atoms that are involved in the hydrogen bond network or the nucleophilic attack of the C‐S bond, as pictured in Figure 2a. The results are shown in Figure S8, validating that HBPS in DszB Ser Glc is positioned similarly to that in WT. Meanwhile, we see no probability density peaks in Figure 6e, suggesting that HBP leaves the active site within the timescale of the simulation, as desired.

Finally, we show in Table 1 the average interaction energy between the substrate (HBPS or HBP) and the protein in each case. We also provide the time evolution of the interaction energy in Figures S9–S10. The total interaction energy confirms that HBPS experiences, on average, a slightly stronger interaction in DszB WT than in the glycosylated systems. However, the average interaction of HBPS with DszB Ser Glc and Thr Glc is still within the first standard deviation range of that in DszB WT such that we would expect similar reactivity profiles. Note that permanent electrostatics dominate the contribution to the total interaction energy for HBPS, as expected. In contrast, van der Waals interactions dominate the contribution to the total interaction energy for HBP, and are lower, on average, for DszB Ser Glc (when in the active site).

**TABLE 1:** Interaction energy between the substrate (HBP or HBPS) and the protein for DszB WT, DszB Ser Glc, and DszB Thr Glc.

In kcal/mol	DszB WT		DszB ser Glc		DszB Thr Glc	
Interaction type	HBPS	HBP	HBPS	HBP	HBPS	HBP
Permanent electrostatics	−141 ± 53	−9 ± 22	−119 ± 48	−5 ± 18	−124 ± 54	−11 ± 21
Induced electrostatics	−20 ± 3	−2 ± 1	−17 ± 4	−1 ± 1	−18 ± 4	−3 ± 2
van der Waals	−18 ± 3	−19 ± 2	−12 ± 4	−13 ± 8	−16 ± 3	−19 ± 2
Total	−179 ± 54	−30 ± 22	−147 ± 49	−19 ± 21	−158 ± 55	−32 ± 22

*Note*: The total interaction energy is the sum of non‐covalent interactions: Permanent electrostatics, induced electrostatics (i.e., polarization), and van der Waals interactions. The numbers are shown as average ± standard deviation.

## 3 CONCLUSION

In summary, we have shown that the selective glycation of Ser residues in the unstructured loops of DszB yields product release within the timescale of our MD simulations while preserving the chemical positioning of the reactant. Product release occurs through a greater conformational flexibility of the loops, generated by the strong hydrophilic properties of glucose. Indeed, the addition of glucose on just three residues generated strong water–loop interactions that were able to overcome the weaker product–loop interactions. Interestingly, when glycation was done on the Thr residues, the gain in conformational flexibility did not yield product release. This is likely due to the fact that only one Thr residue was glycated in loop 3, the largest loop, compared to two Ser residues. This would suggest that the glycation of residues needs to be spread out throughout the loops according to their length. Overall, among the three loops, loop 3 was observed to be the most flexible, while loop 1 was the least affected by glycation.

Our computational study suggests that selective glycation is a valid strategy to moderately enhance the conformational flexibility in DszB. This increased conformational flexibility facilitates product release as also reported by Han et al. for another enzyme (Han et al., 2016) Our strategy has the added advantage that posttranslational modifications do not modify the chemical structure of the product binding site; therefore, they do not modify the chemical structure of the reactant binding site. Future works will involve testing the sensitivity of the procedure to the nature and number of the glycated residues.

## 4 METHODS

In this paper, we performed molecular dynamics (MD) simulations with the polarizable AMOEBA force field (Laury et al., 2015; Ponder et al., 2010).

We prepared our MD input files from the crystal structure of HBPS‐bound DszB (PDB ID 2DE3) (Berman et al., 2000). The last 17 residues whose structures were not resolved and formed an unstructured tail were removed to keep 347 residues. We generated 25 backbone conformations with the backrub (Davis et al., 2006; Smith & Kortemme, 2010) application of rosetta (Simons et al., 1999). We picked the 3 lowest energy conformers out of the 25 and repacked the side chains with fixbb (Dantas et al., 2003; Shapovalov & Dunbrack, 2011). These 3 DszB conformations were used to run 3 independent MD replicates characterizing substrate‐bound DszB WT. The apo DszB WT was modeled by deleting HBPS. The product‐bound DszB WT was modeled by deleting the SO_2_^−^ group in HBPS to form HBP on all three replicates. We then duplicated the three apo, ligand‐ and product‐bound conformers and attached glucose on all Ser or Thr residues in loop 1, loop 2, and loop 3. Loop 1 spans residues 50 to 60, loop 2135 to 150, and loop 3180 to 200. Accordingly, the residues that were attached to glucose were Ser53, Ser181, and Ser194 in the case of the serine‐glycated DszB (DszB Ser Glc) and Thr58, Thr148, and Thr196 in the case of the threonine‐glycated DszB (DszB Thr Glc). For comparison, we also modeled the apo state from PDB ID 2DE3, one replica for DszB WT, DszB Ser Glc, and DszB Thr Glc.

We solvated each system (30 in total) in a 90×90×90 Å^3^ cubic water box using the xyzedit (Rackers et al., 2018) executable of the tinker software (Wang, 2021) package. Finally, we balanced the charge of the protein with sodium and chloride ions. The input files are provided in our group's GitHub. AMOEBA parameters for glucose, HBP, and HBPS were found through poltype2 (Walker et al., 2022) and provided in our group's GitHub. The parameters for DszB, water, and the ions were taken from the amoebabio18 parameter file (Laury et al., 2015). We first ran an energy minimization on each of the 30 systems, using limited memory Broyden–Fletcher–Goldfarb–Shanno (L‐BFGS) algorithm with an RMS gradient of 0.1 kcal/mol/Å.

MD simulations were run on the minimized structures under a constant number of atoms, pressure (1 atm), and temperature (300 K) with the Tinker9 package (Wang, 2021). Each MD was 150 ns in total with a 1 fs time step. Root mean square deviation (RMSD) and RMSF were calculated using mdanalysis (Gowers et al., 2019; Michaud‐Agrawal et al., 2011). The reference frame used for the RMSD is the initial frame of the MD simulation, measuring the average deviation of the amino acids over time (Figures S11 and S12). The reference frame used for the RMSF is the average structure in the simulation, quantifying the average fluctuation of each residue in the simulation. The convergence of the RMSF plot is shown in Figure S13. The initial 50 ns were taken as equilibration and each property was then calculated as the average of the 3 replicate MDs, for a total production time of 300 ns. This includes the ψ‐ϕ angle pairs (computed with mdanalysis), the HBPS‐loop, HBP‐loop, HBPS‐active site residues, HBP‐active site residues, and loop‐active site residue distances reported in the main text. These distances were computed with a python script that extracted the coordinates of each carbon alpha in the loop residue as well as the center of mass of the substrate and active site residues. The reported distances are then the averaged pairwise distances in each case. The convergence for the distributions shown in the main text is shown in Figures S14 and S15. Finally, the secondary structure of each loop during the simulations is given in Figures S16–S18, confirming the unstructured nature of these segments when unbound.

## AUTHOR CONTRIBUTIONS

**Junbao Liang:** Writing – original draft; investigation; writing – review and editing; visualization; methodology; validation; data curation; formal analysis. **Yi Zheng:** Investigation; methodology; formal analysis. **Valerie Vaissier Welborn:** Conceptualization; funding acquisition; writing – original draft; writing – review and editing; project administration; supervision; resources; methodology.

## FUNDING INFORMATION

Petroleum Research Fund of the American Chemical Society, Grant/Award Number: 66767‐ND6.

## CONFLICT OF INTEREST STATEMENT

There are no conflicts to declare.

## Supporting information


**Data S1.** Supporting Information.

## Data Availability

The data that support the findings of this study are openly available in proteinscience_DszB at https://github.com/WelbornGroup/proteinscience_DszB.git.
