# Supplementary material for: Mitigating product inhibition in 2′‐hydroxybiphenyl‐2‐sulfinate desulfinase (DszB) with synthetic glycosylation
Source: Protein Sci. 2025 Jun 16;34(7):e70187. doi: 10.1002/pro.70187 (PMC12168087; doi:10.1002/pro.70187)
Supplement: Supplementary file 1 — Data S1. Supporting Information. [file PRO-34-e70187-s001.pdf]

Mitigating product inhibition in 2'-hydroxybiphenyl-2-sulfinate desulfinase (DszB) with synthetic glycosylation

Junbao Liang,† Yi Zheng,† and Valerie Vaissier Welborn*,†,‡

†*Department of Chemistry, Virginia Tech, Blacksburg, VA 24060, USA*

‡*Macromolecules Innovation Institute (MII), Virginia Tech, Blacksburg, VA 24060, USA*

E-mail: vwelborn@vt.edu

MD input file availability

Poltype 2 and Tinker 9 input with parameters files are available at https://github.com/WelbornGroup/.

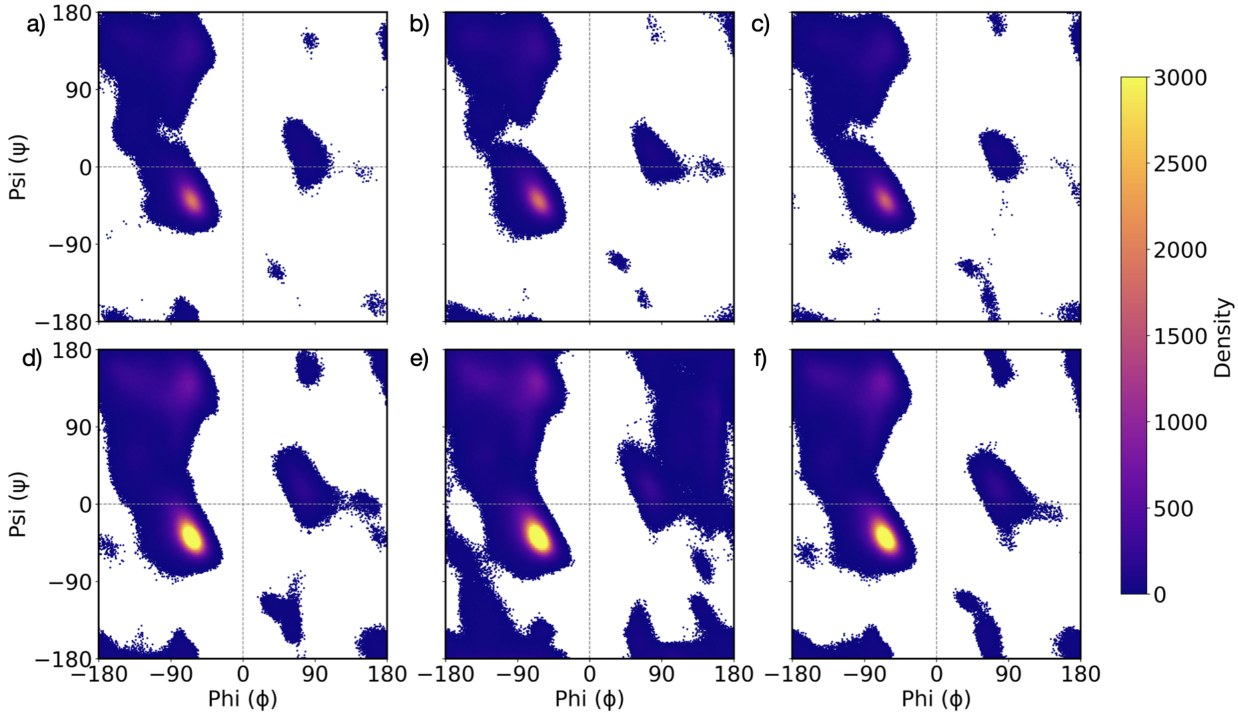

Figure S1: Ramachandran plots showing the distribution of the (ψ, ϕ) dihedral pairs in a) DszB WT in the apo state (PDB ID: 2de2), b) DszB Ser Glc in the apo state (PDB ID: 2de2), c) DszB Thr Glc in the apo state (PDB ID: 2de2), d) DszB WT in the apo state (PDB ID: 2de3), e) DszB Ser Glc in the apo state (PDB ID: 2de3), f) DszB Thr Glc in the apo state (PDB ID: 2de3).

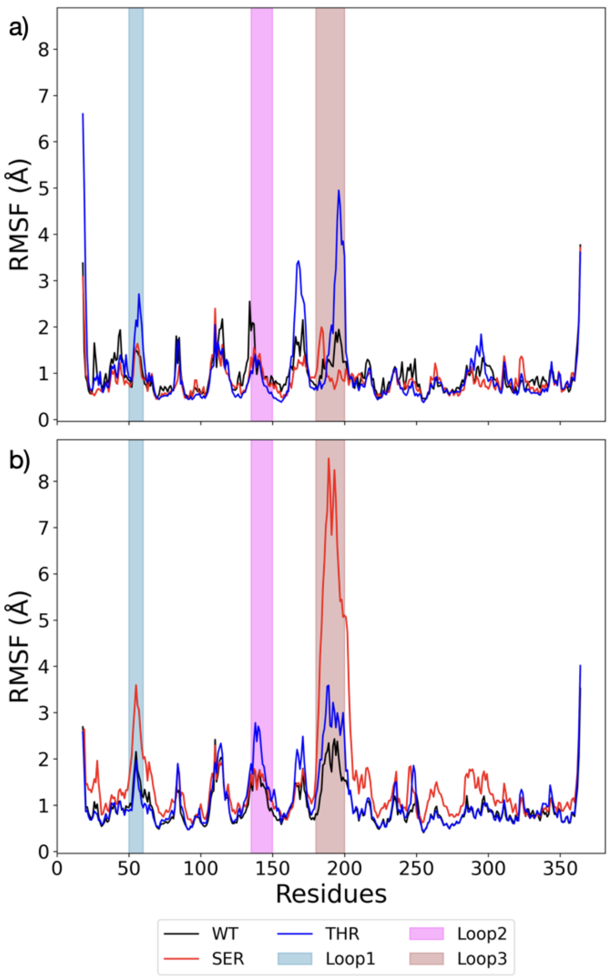

Figure S2: RMSF calculated for DszB WT, Ser Glc and Thr Glc in the a) apo state (PDB ID: 2de2), b) apo state (PDB ID: 2de3). The residue index range corresponding to the three loops obstructing the active site in DszB is shown in blue (loop 1), magenta (loop 2) and brown (loop 3).

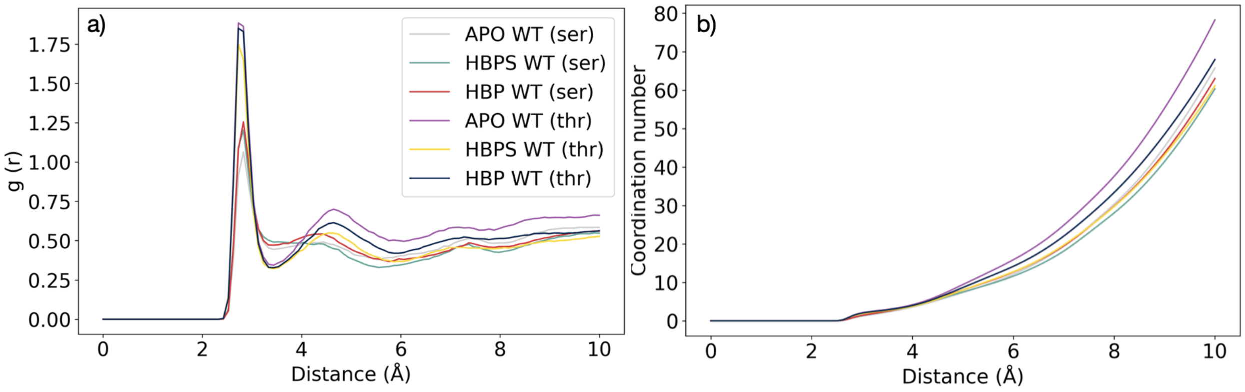

Figure S3: a) Radial pair distribution function and b) its integral between the O of the Thr and Ser residues linked to glucose and the O of water for DszB WT.

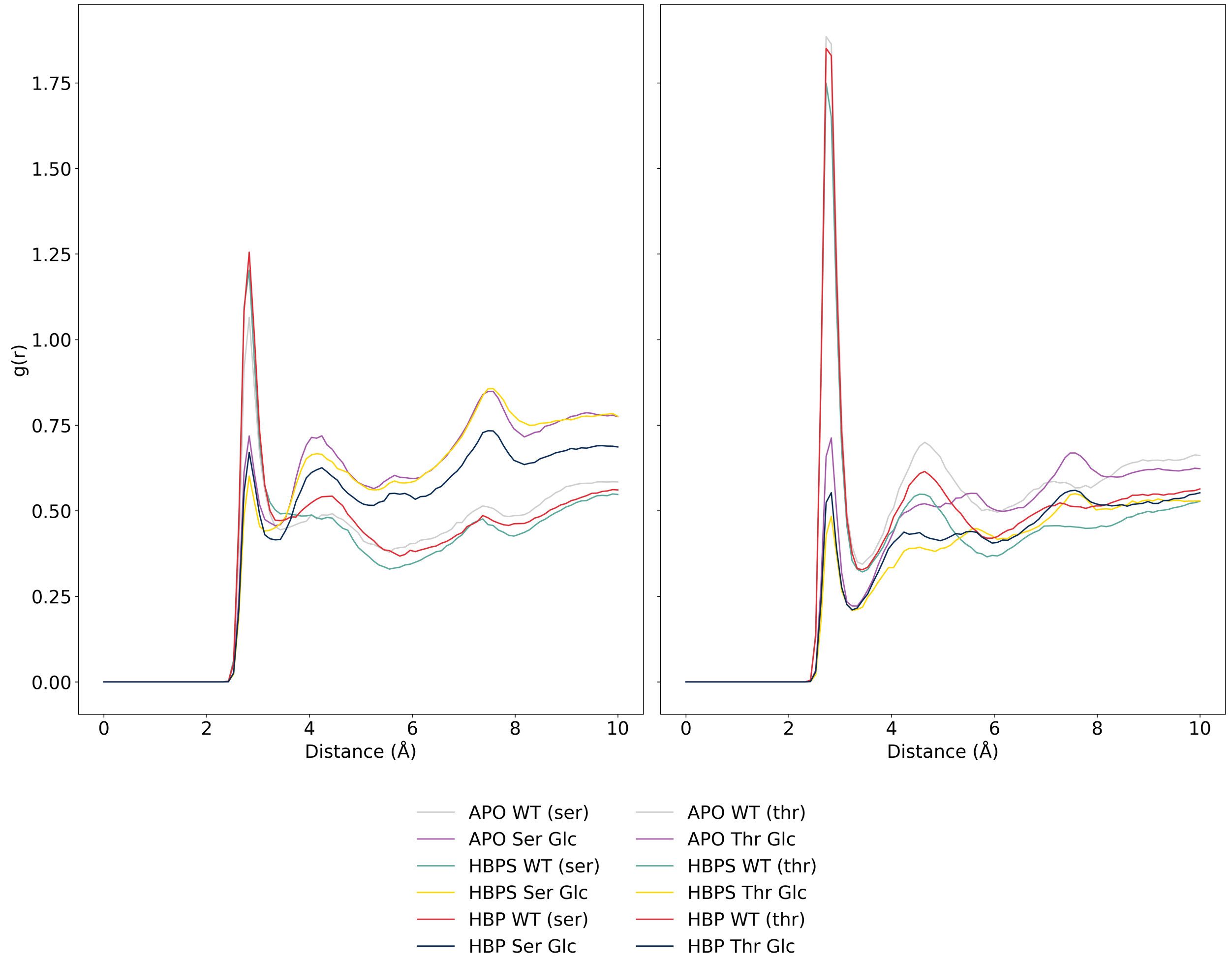

Figure S4: Radial Pair Distribution function between the O of the Thr and Ser residues linked to glucose and the O of water for DszB WT, Ser Glc, and Thr Glc.

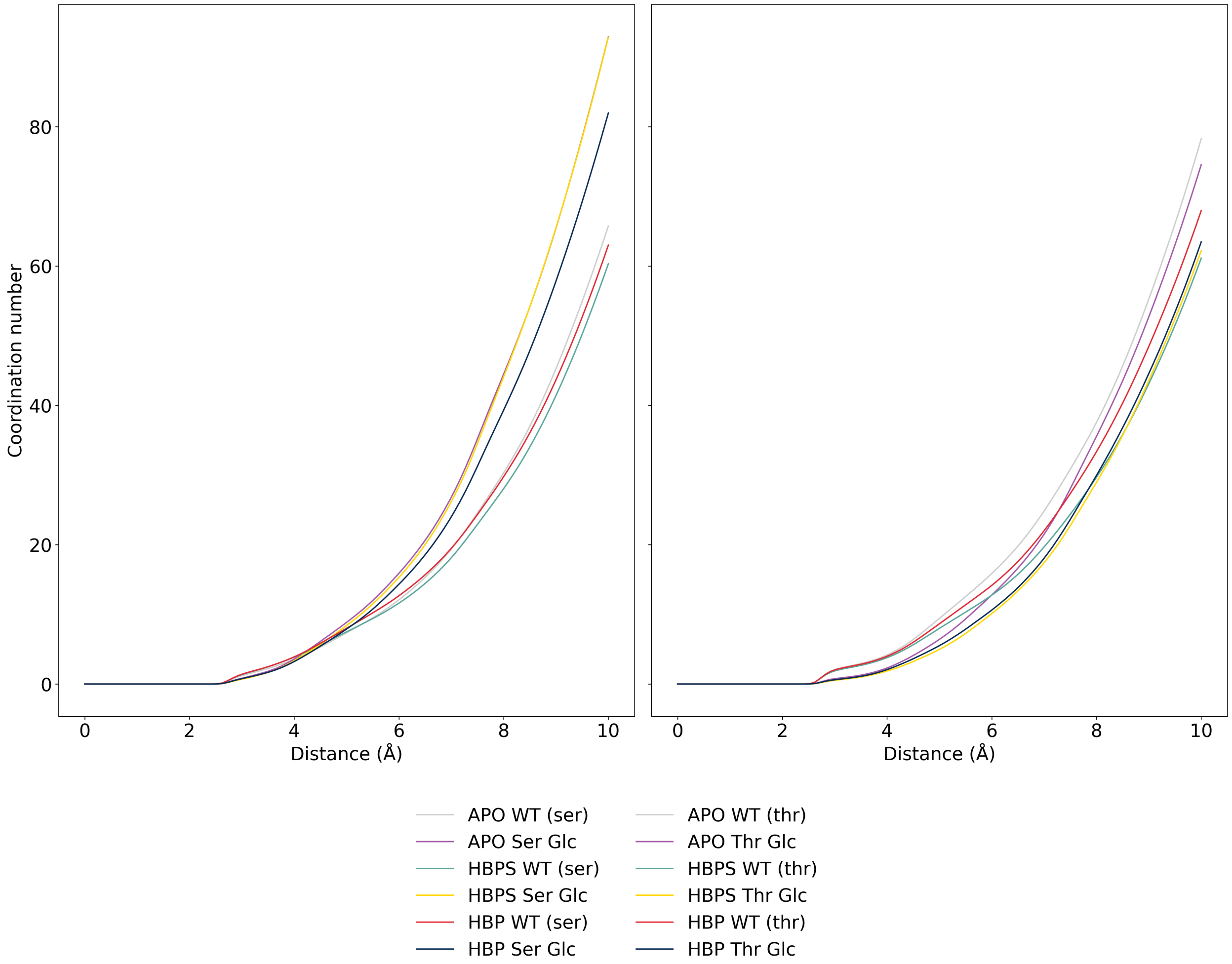

Figure S5: Water coordination number of the Thr and Ser residues in DszB WT, Ser Glc, and Thr Glc, computed as the integral of the radial distribution function in Figure S4.

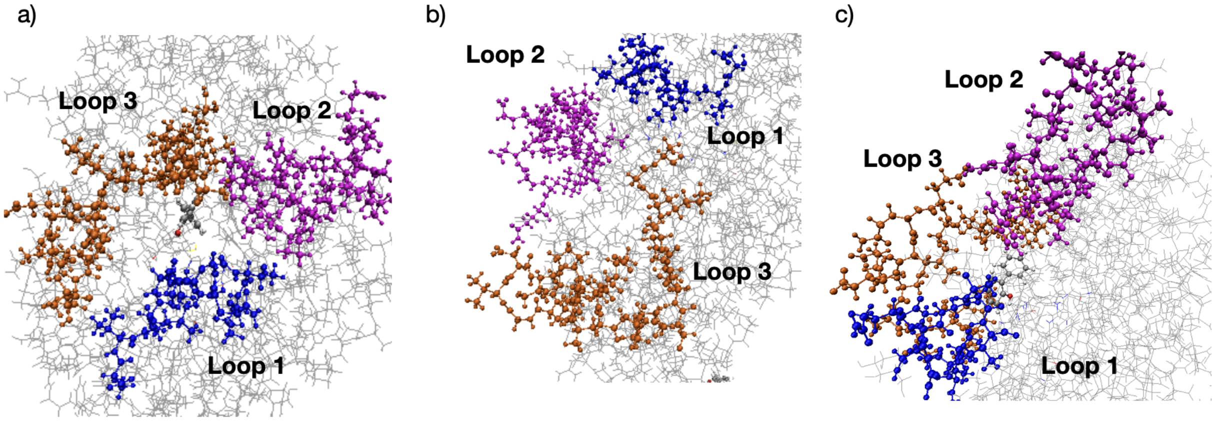

Figure S6: The loop flexibility comparison between WT, Ser Glc, and Thr Glc in product (HBP)-bound state in one of the simulation. The snapshots are taken in the last frame of 150 ns trajectory. a) DszB WT, b) DszB Ser Glc, and c) DszB Thr Glc. The rest of the snapshots are available in our group's Github.

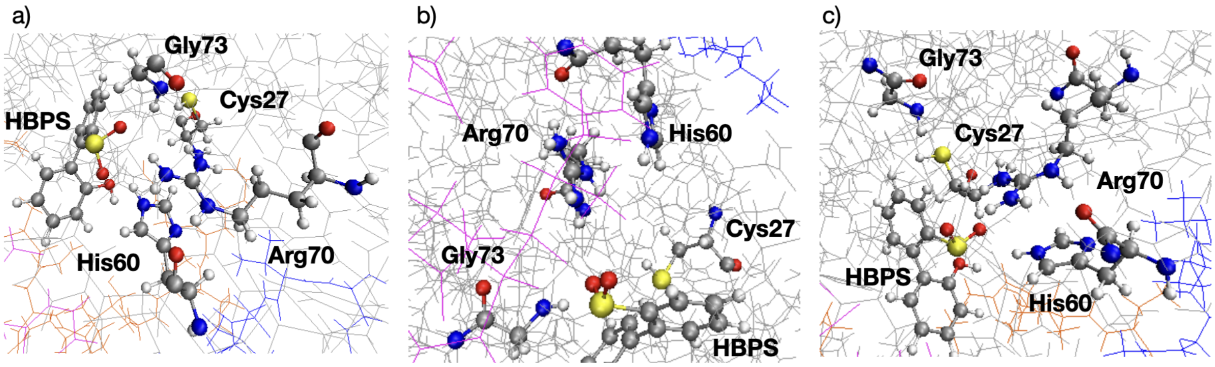

Figure S7: The chemical positioning comparison between WT, Ser Glc, and Thr Glc in substrate (HBPS)-bound state in one of the simulation. The snapshots are taken in the last frame of 150 ns trajectory. a) DszB WT, b) DszB Ser Glc, and c) DszB Thr Glc. The rest of the snapshots are available in our group's Github.

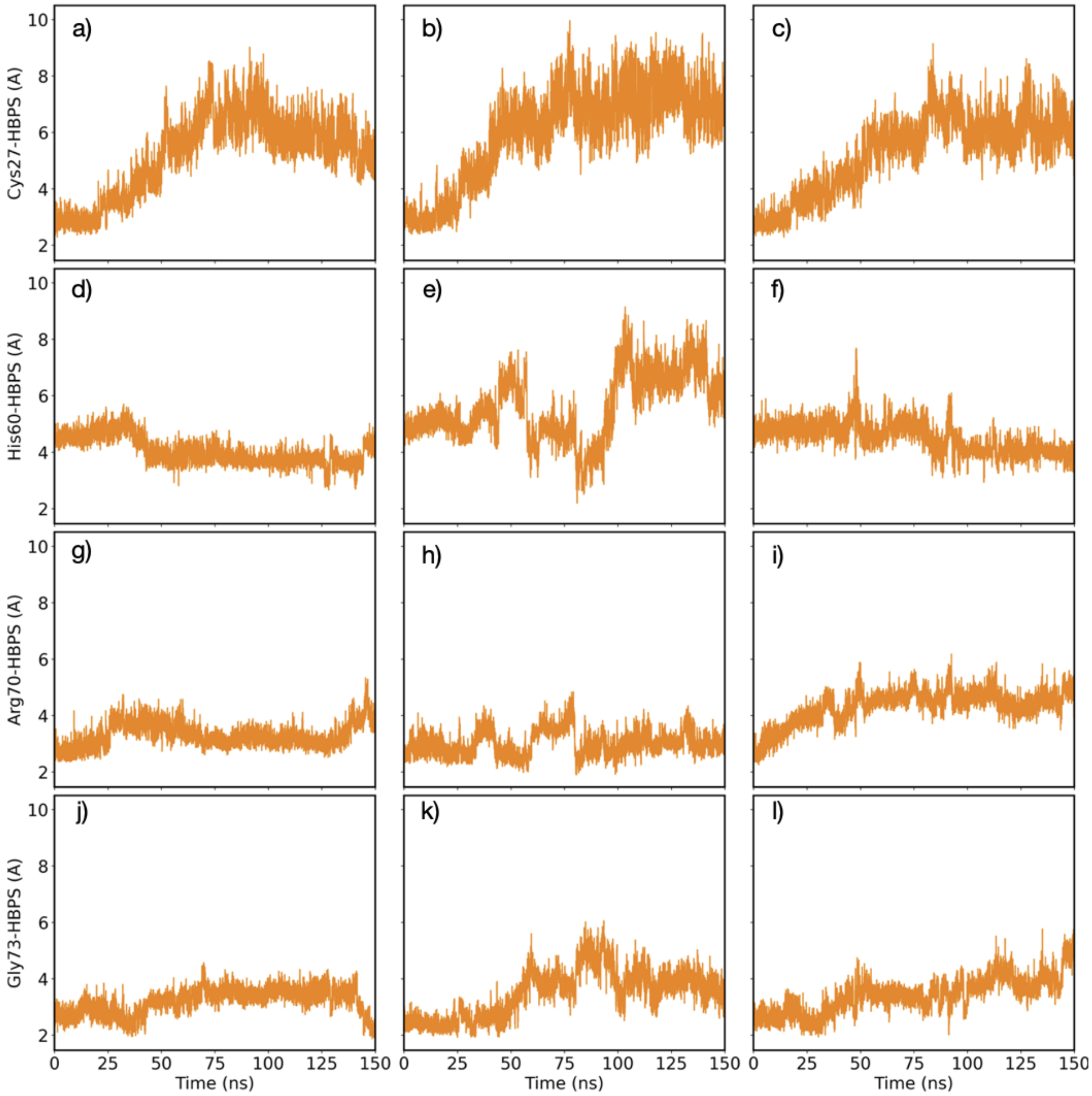

Figure S8: Chemical positioning of HBPS in the active site of DszB WT, Ser Glc and Thr Glc. Distance between the hydrogen of HS in Cys27 and the oxygen of SO_2 in HBPS for a) DszB WT, b) DszB Ser Glc and c) DszB Thr Glc. Distance between the hydrogen in His60 and the oxygen of SO_2 in HBPS for d) DszB WT, e) DszB Ser Glc and f) DszB Thr Glc. Distance between the hydrogen in Arg70 and the oxygen of SO_2 in HBPS for g) DszB WT, h) DszB Ser Glc and i) DszB Thr Glc. Distance between the hydrogen in Gly73 and the oxygen of SO_2 in HBPS for j) DszB WT, k) DszB Ser Glc and l) DszB Thr Glc.

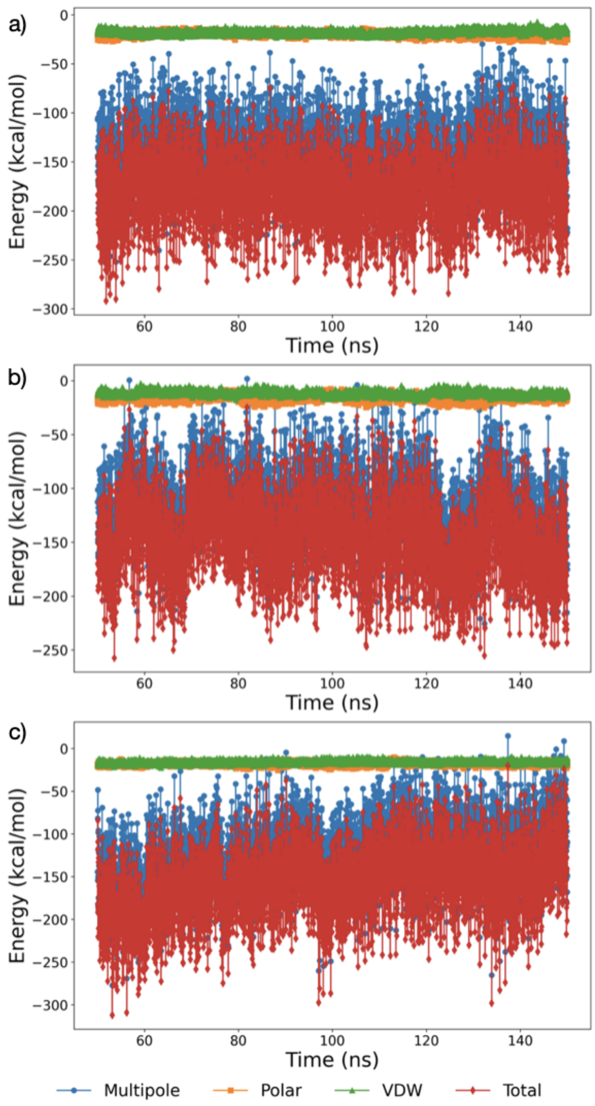

Figure S9: Interaction energy between substrate HBPS and DszB. a) DszB WT, b) DszB Ser Glc, c) DszB Thr Glc

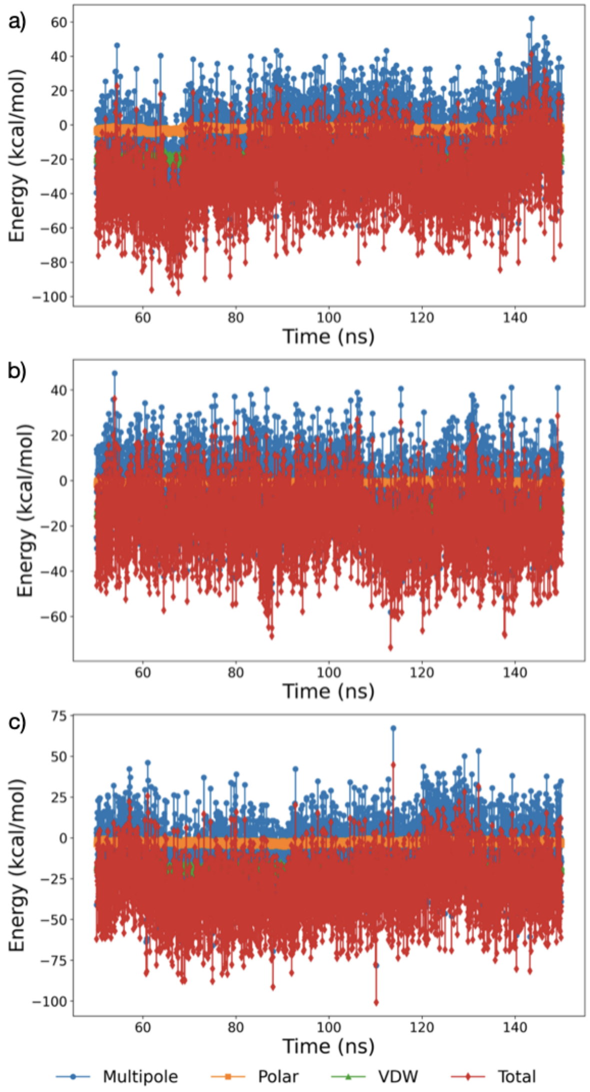

Figure S10: Interaction energy between substrate HBP and the protein. a) DszB WT, b) DszB Ser Glc, c) DszB Thr Glc

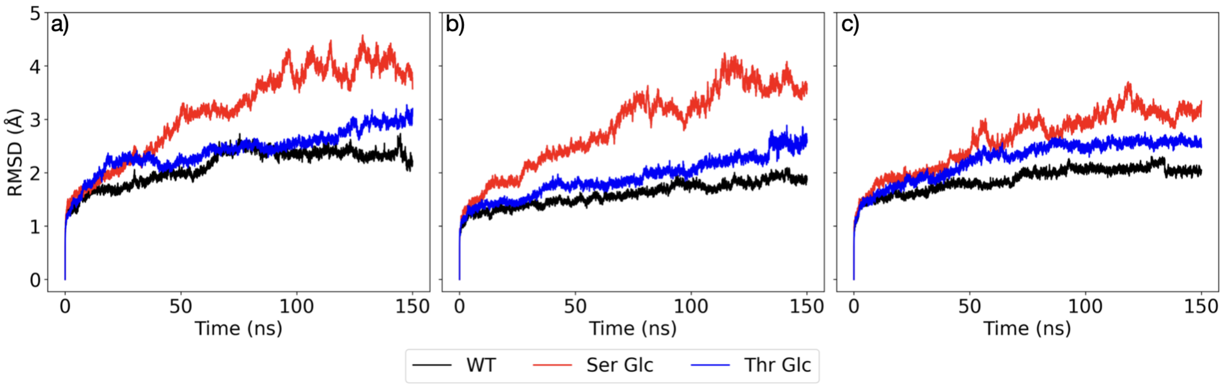

Figure S11: Root mean square deviation (RMSD) of the protein backbone as a function of simulation time for DszB WT, Ser Glc and Thr Glc in the a) apo state, b) substrate (HBPS)-bound state and the c) product (HBP)-bound state.

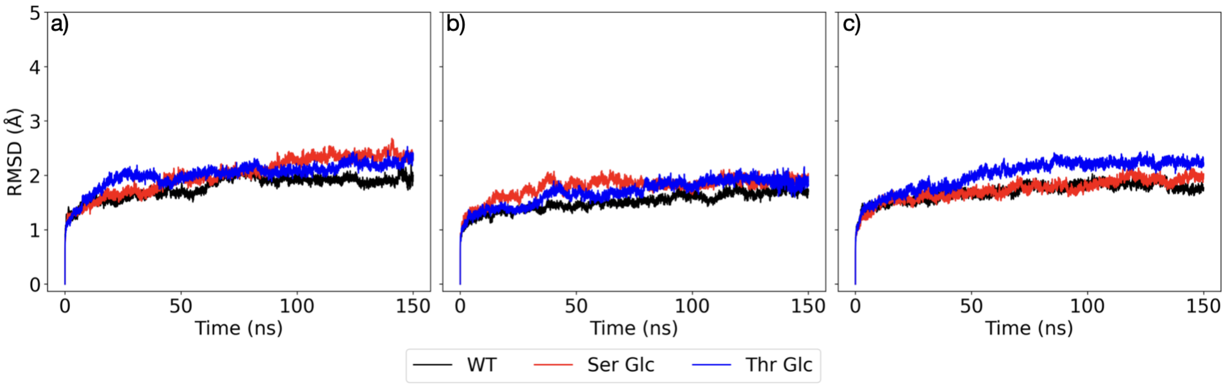

Figure S12: Root mean square deviation (RMSD) of the protein backbone without the unstructured loops as a function of simulation time for DszB WT, Ser Glc and Thr Glc in the a) apo state, b) substrate (HBPS)-bound state and the c) product (HBP)-bound state.

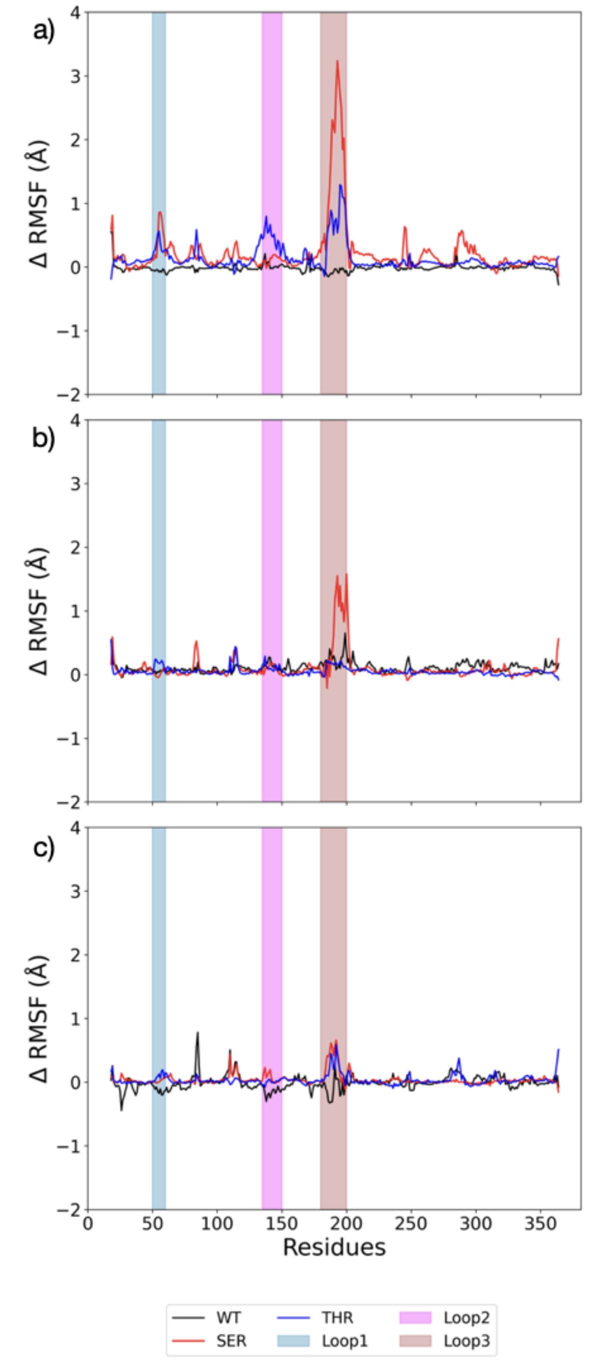

Figure S13: Difference in RMSF calculated over the initial 120 ns and 150 ns trajectories. a) DszB WT, b) DszB Ser Glc, c) DszB Thr Glc

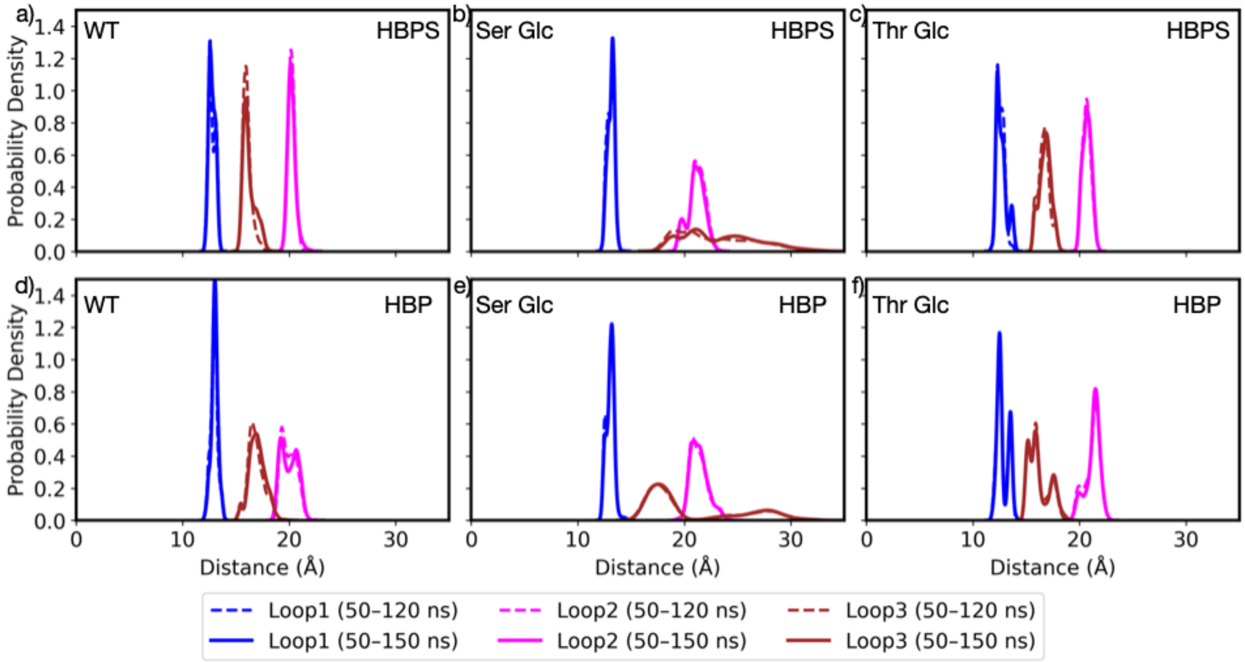

Figure S14: Comparing initial (50-120 ns) and extended (50-150 ns) simulation of probability density of the average pairwise distance between the residues in the loops and the active site residues Cys27, Arg70, Gly73 and His60 in a) DszB WT in the ligand (HBPS)-bound state, b) DszB Ser Glc in the ligand (HBPS)-bound state, c) DszB Thr Glc in the ligand (HBPS)-bound state, d) DszB WT in the product (HBP)-bound state, e) DszB Ser Glc in the product (HBP)-bound state, f) DszB Thr Glc in the product (HBP)-bound state.

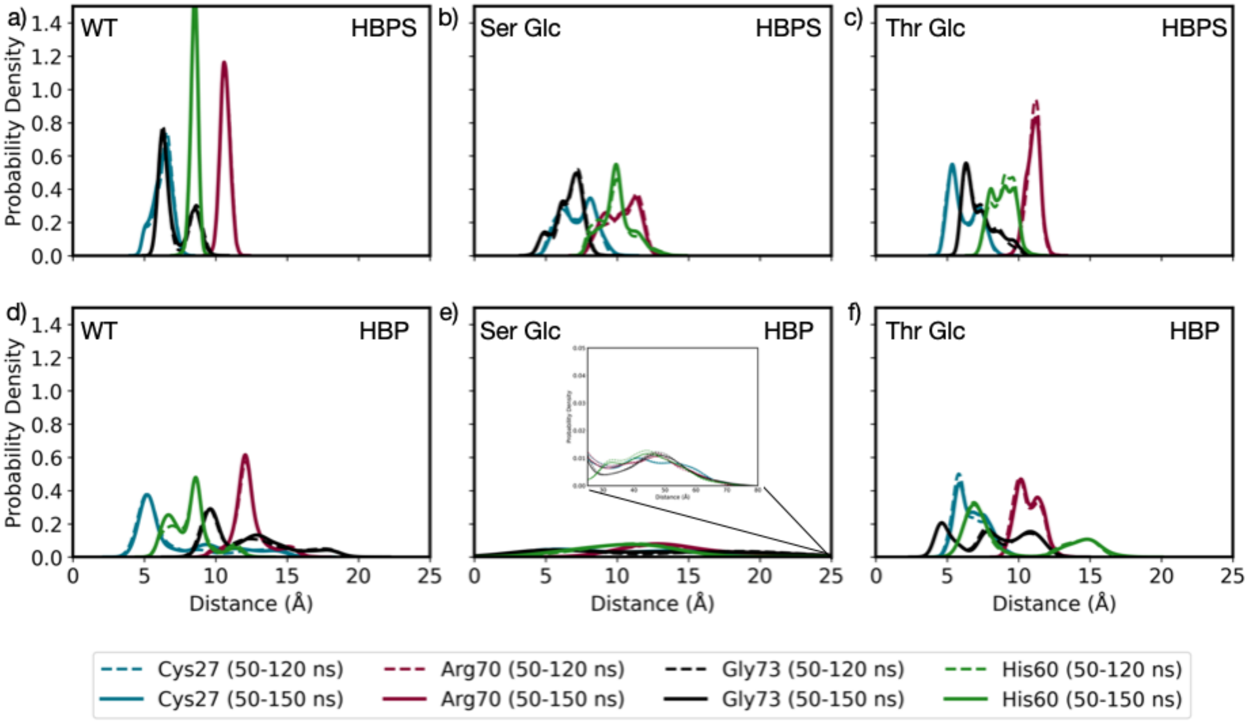

Figure S15: Comparing initial (50-120 ns) and extended (50-150 ns) simulation of probability density of the average pairwise distance between the substrate (HBPS or HBP) and the active site residues Cys27, Arg70, Gly73 and His60 in a) DszB WT in the ligand (HBPS)-bound state, b) DszB Ser Glc in the ligand (HBPS)-bound state, c) DszB Thr Glc in the ligand (HBPS)-bound state, d) DszB WT in the product (HBP)-bound state, e) DszB Ser Glc in the product (HBP)-bound state, f) DszB Thr Glc in the product (HBP)-bound state.

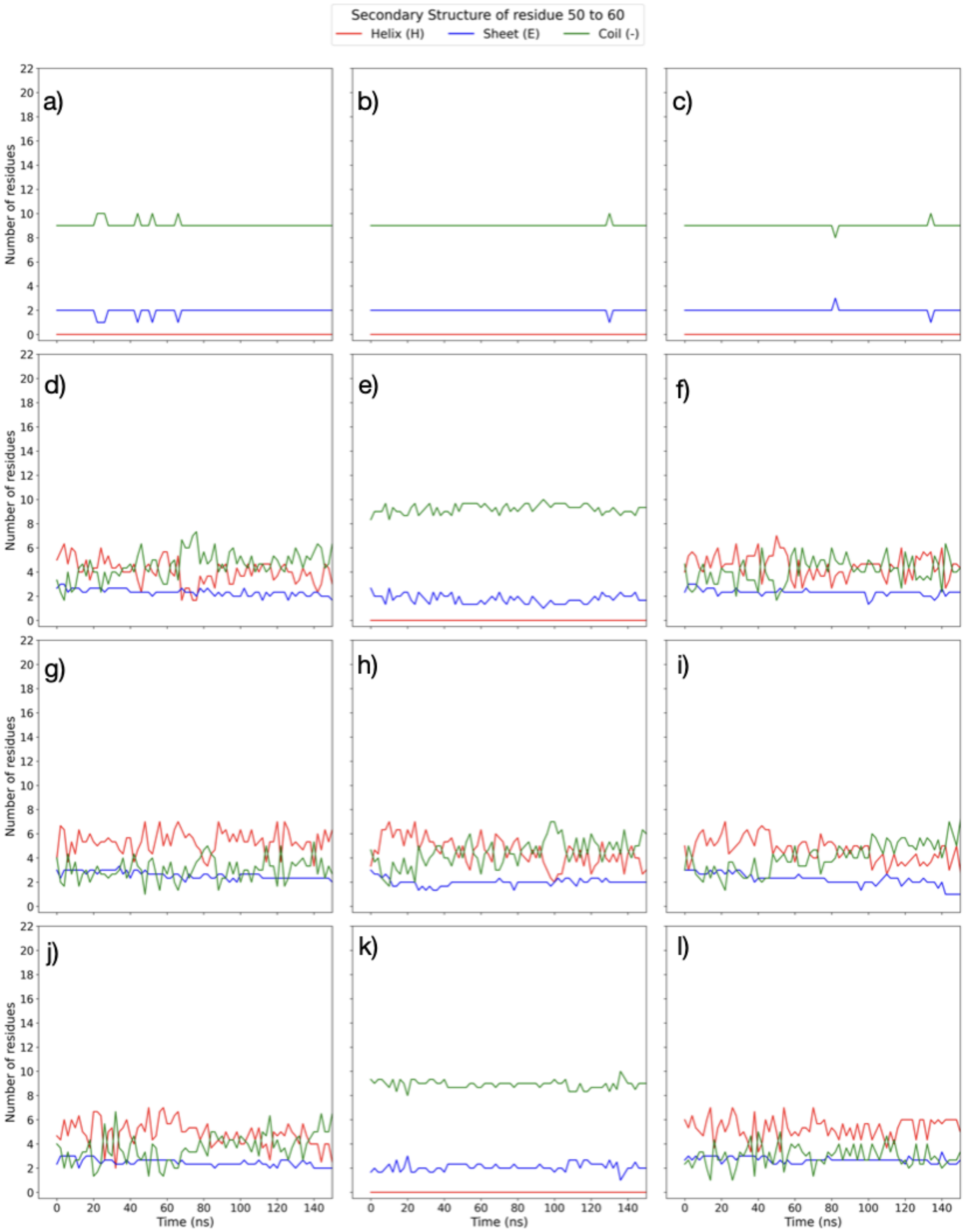

Figure S16: Secondary structure analysis of loop1 residues using DSSP module in MDAnalysis. apo state (PDB id: 2de2): a) DszB WT, b) DszB Ser Glc, and c) DszB Thr Glc. apo state (PDB id: 2de3): d) DszB WT, e) DszB Ser Glc, and f) DszB Thr Glc. substrate (HBPS)-bond state: g) DszB WT, h) DszB Ser Glc, and i) DszB Thr Glc. product (HBP)-bound state j) DszB WT, k) DszB Ser Glc, and l) DszB Thr Glc.

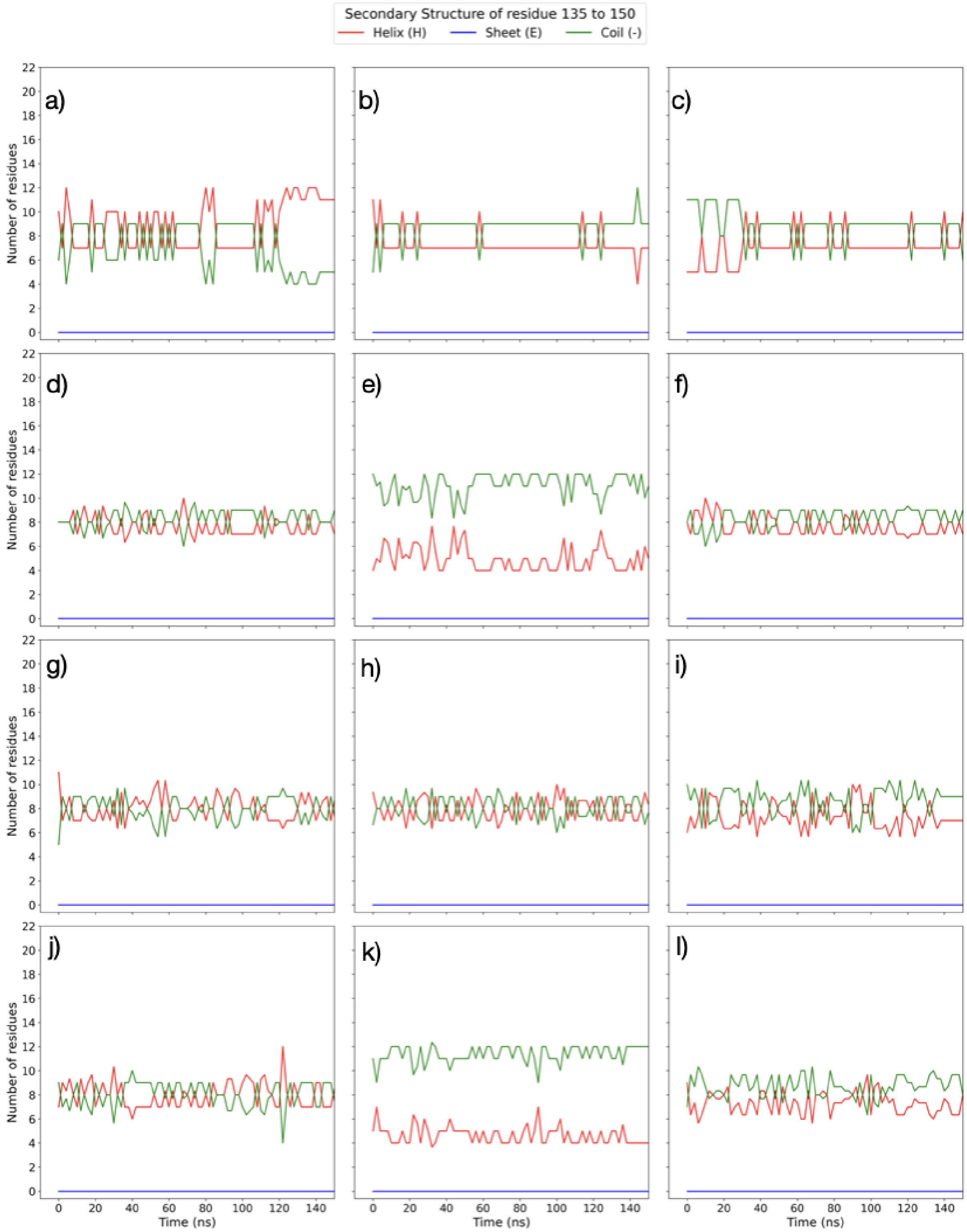

Figure S17: Secondary structure analysis of loop2 residues using DSSP module in MDAnalysis. apo state (PDB id: 2de2): a) DszB WT, b) DszB Ser Glc, and c) DszB Thr Glc. apo state (PDB id: 2de3): d) DszB WT, e) DszB Ser Glc, and f) DszB Thr Glc. substrate (HBPS)-bond state: g) DszB WT, h) DszB Ser Glc, and i) DszB Thr Glc. product (HBP)-bound state j) DszB WT, k) DszB Ser Glc, and l) DszB Thr Glc.

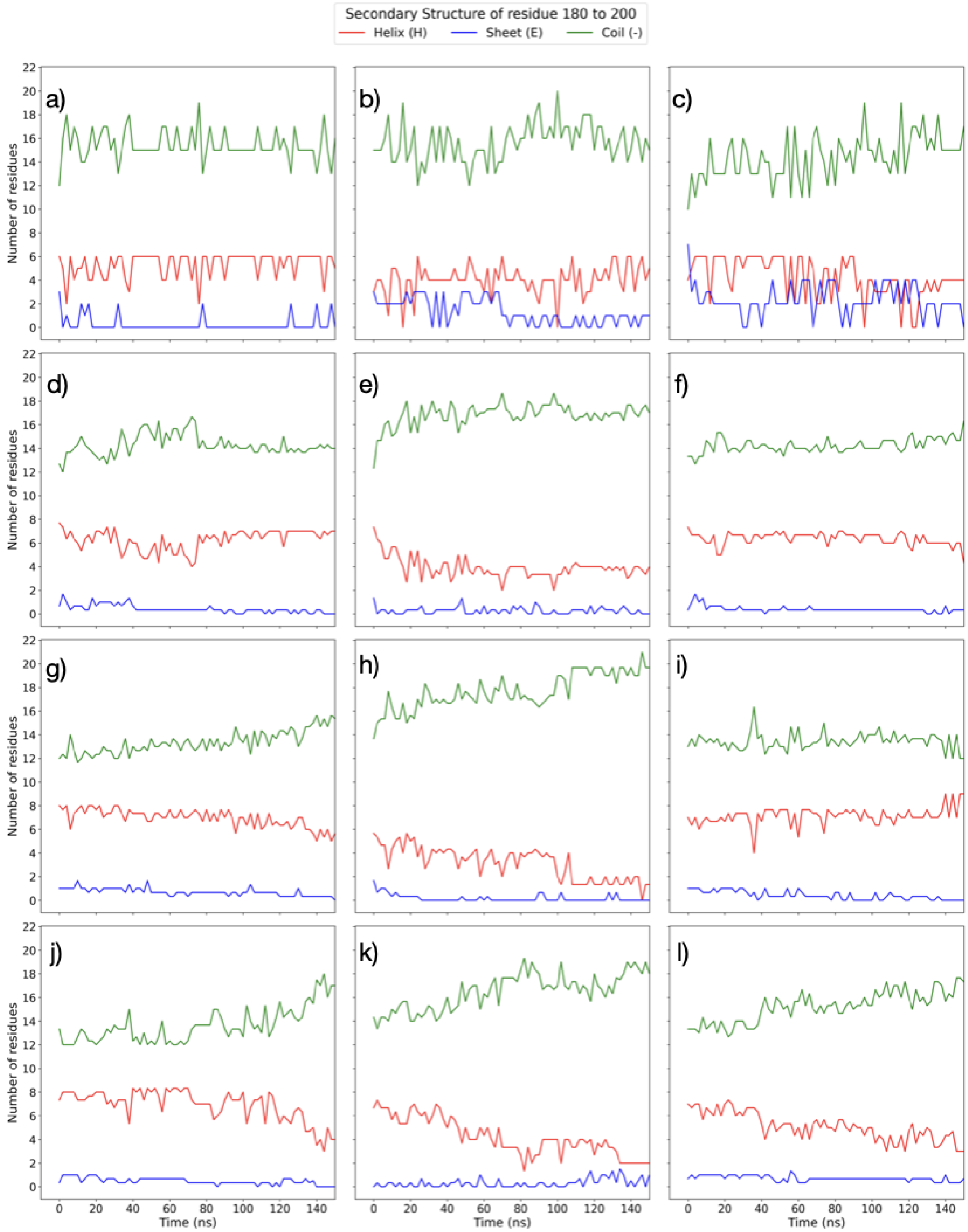

Figure S18: Secondary structure analysis of loop3 residues using DSSP module in MDAnalysis. apo state (PDB id: 2de2): a) DszB WT, b) DszB Ser Glc, and c) DszB Thr Glc. apo state (PDB id: 2de3): d) DszB WT, e) DszB Ser Glc, and f) DszB Thr Glc. substrate (HBPS)-bond state: g) DszB WT, h) DszB Ser Glc, and i) DszB Thr Glc. product (HBP)-bound state j) DszB WT, k) DszB Ser Glc, and l) DszB Thr Glc.
